# Cocaine in Surgery

**Published:** 1893-10

**Authors:** R. H. Cowan


ARTICLE IV.

### COCAINE IN SURGERY.

BY R. H. COWAN.

While ether and chloroform are in daily use as anæsthetics and reports of death from either of these agents are rare, yet in the hands of the most careful and experienced fatal narcosis does occasionally occur. In a larger proportion of cases alarming symptoms are present, sometimes followed by permanent bad results. Of course, in common with other surgeons, I recognize in these anæsthetics a wonderful boon both to patient and operator; indeed, I can hardly understand how the surgeons of the past got along without them; and I think this feeling must be shared by others. I can but look forward with impatient anxiety to the discovery of some agent, which, while equally efficacious in allaying pain, can claim the superior merit of *absolute safety.*

When cocaine was first introduced to the profession, and exaggerated reports of its wonderful properties were published, I looked with much interest for report of its use, and availed myself of the first opportunity to employ it in operations on the eye and later in minor operations.

Within the last twelve months I have ventured farther and performed several more serious operations with cocaine as my anæsthetic, and in every instance its action has been all I could desire. Anæsthesia has been perfect, no bad symptoms have occurred, and union by first intention has been the rule.

The operations have been as follows: A large and deeply imbedded tumor (adipose) removed from the popliteal region, and seven amputations—four of the leg, and one each of the thigh, forearm and arm.

Cocaine was, I believe, first advised in amputations by Dr. Corning, and his advice was strengthened by an actual experience. Why he has not had more followers I do not know; it is, however, for this very reason that I am induced to contribute my meager experience. I am well aware that we have reports of disastrous results from cocaine, nor would I countenance the reckless administration of a drug with whose properties we are as yet but little acquainted.

Whether or not cacaine will supersede ether and chloroform in the near future I cannot say; but believing it is only by reports from actual experience that we can arrive at any definite knowledge of its virtues, I desire to contribute my mite.

Before concluding, I may mention some of the advantages which, it seems to me, are secured by the use of cocaine:

1. Absence of depressing effects in cases of severe shock; or of constitutional weakness.

2. Freedom from nausea and vomiting after operations.

3. Limitation of anæsthesia (of course constriction with an Esmarch above point of operations is made) to the field of operation, and consequent comparative security from fatal narcosis.

In the above-mentioned operations (with the exception of the two first amputations) I have, at the suggestion of Dr. Wyeth, employed a two per cent. solution. This strength, while proving equally or perhaps more efficient in producing anæsthesia, possesses the additional advantage of reducing to a minimum the danger of any toxic effect.—*International Journal of Surgery.*

